# Tool to assess recognition and understanding of elements in Summary of Findings Table for health evidence synthesis: a cross-sectional study

**DOI:** 10.1038/s41598-023-45359-x

**Published:** 2023-10-23

**Authors:** Jakov Matas, Ružica Tokalić, Daniel García-Costa, Emilia López-Iñesta, Elena Álvarez-García, Francisco Grimaldo, Ana Marušić

**Affiliations:** 1 Department of Research in Biomedicine and Health, Center for Evidence-Based Medicine, University of Split School of Medicine, Šoltanska 2, 21000 Split, Croatia; 2 Computer Science Department, Universitat de València, Valencia, Spain; 3 Department of Didactics of Mathematics, Universitat de València, Valencia, Spain

**Keywords:** Medical research, Epidemiology, Clinical trial design

## Abstract

Summary of Findings (SoF) tables concisely present the main findings of evidence synthesis of health evidence, but how users navigate it to understand and interpret the presented information is not clear. We quantified the interaction of medical students with an SoF table while answering a knowledge quiz. Read&Learn tool was used to measure the number of target and non-target table cells visited for each question and the time spent on these cells. Students positively identified target elements for quiz questions and answered simpler questions, but struggled with critical thinking and understanding study outcomes. The question on outcomes with the largest improvement post-intervention had the fewest correct answers, the longest interaction with table cells and the most opened cells before answering. Students spent a median of 72% of the time reading target table cells. A heatmap of the interactions showed that they were mostly answer-oriented. Further development of the tool and metrics is needed to use the tool and the metrics to study the cognitive processes during the assessment of health evidence.

## Introduction

A ‘Summary of Findings’ (SoF) table presents the main findings in evidence synthesis of health research results, structured in a simple, standardized table, making it easier for a reader to understand and critically appraise the evidence^1^. SoF tables have been studied to assess how they affect understanding and retrieval of a review’s key findings^2,3^, showing that the inclusion of an SoF table improved the understanding and rapid retrieval of key study findings, compared to studies without an SoF table. The structure of the SoF table has been optimized to make it more suitable for clinical audiences^4,5^.

An important issue for SoF table users is how to navigate it in order to understand and interpret the information. This is especially relevant when the evidence from an SoF table is used for decision-making in health practice. There are different theories about how humans focus their attention in large visual field^6^, such as over 50 table cells in an SoF table. The interaction of the reader and the table in a cognitive task of looking for an answer to a knowledge question can be tested in different ways and tools, including eye tracking and hand/finger tracking^7^. These tools have the limitation that they are only a proxy for cognitive attention, as the movements of the eyes or fingers many not correlate with the cognitive processes (mind wondering) or may be influenced by the presentation of the visual stimuli^8^. In this study, we used a novel software Read&Learn tool^9^, developed for analysing the process of navigation in a written educational task. The tool quantifies reading comprehension of a student by computing the times the segments of a text or textual structures are visited before answering the question.

The objective of our study was to quantify students’ interaction with and SoF table when they answer quiz questions related to the health evidence presented in the table. In the experiment, the students who have passed courses in research methodology and evidence-based medicine^10^, including the basing information about assessing the quality of evidence and SoF table, were presented by a SoF table on the effects of an antiviral drug vs standard care on the clinical status of patients with moderate COVID-19^11^. All the SoF table cells were blurred, except the row and column headings, and students had to open cells in order to answer knowledge test questions about the information in the SoF table. We recorded which table cells were visited, whether they were relevant for the question asked and the total time spent on cells for different quiz questions.

## Results

We analysed course quiz results from 121 students, 80 from the Croatian study program (92% attendance) and 41 from the English study program (60% attendance). They correctly answered a median of 4 out of 6 questions (Table 1). The total time spent on the quiz was around 14 min, with very little time spent on the initial reading, i.e. opening the quiz and reading before opening the first question. Overall, a median of 87 segments were visited by a student during the quiz (one table segment could be opened more than once). Very few segments were visited during the initial reading. For questions which had target segments (questions Q2 and Q4 to Q7), the students spent a median of 72% (interquartile range (IQR) = 54–86%) of the time reading the target table cells. There were no significant differences between the students from the English and the Croatian study programme in reading times and segments visited (Table 2). Overall, the students from the Croatian program had significantly more correct answers than those from the English program (Table 2).

**Table 1:** Number of correct answers, number segments visited and times spent (median, interquartile range) on Summary of Findings Table^a^.

Parameter	Total (n = 121)	English program students (n = 41)	Croatian program students (n = 80)	*P* value
No. correct answers^b^	4 (3–4)	3 (2–4)	4 (3–5)	0.002
Total time (seconds) spent on the quiz, from the beginning to the end	833.6 (652.0–980.2)	821.3 (632.4–978.8)	811.9 (671.4–978.0)	0.965
Time (seconds) spent on initial reading^c^	0 (0–205.6)	0 (0–9.4)	0 (0–286.2)	0.270
Time (seconds) spent on quiz questions	355.0 (277.7–472.5)	392.9 (294.2–470.6)	343.3 (270.0–475.5)	0.715
Total number of segments visited	87 (54–129)	84 (50–109)	92 (55.7–134)	0.030
Numbers of segments visited during the initial reading	0 (0–3)	1 (0–3)	0 (0–2.5)	0.080
Number of segments visited during answering the quiz questions	87.5 (54.75–129)	84 (50–109)	94 (57.5–134)	0.107
Total time (seconds) spent on reading target segments	465.4 (280.1–602.1)	380.5 (246.6–533.5)	478.3 (302.3–614.0)	0.115
Time (seconds) spent on reading target segments during initial reading	0.0 (0.0–21.5)	0.0 (0.0–9.4)	0.0 (0.0–8.8)	0.102
Time (seconds) spent on reading target segments during answering the quiz questions	426 (259.9–588.3)	380.5 (240.8–524.5)	467.9 (278.0–613.8)	0.164
Total time (seconds) spent on reading non-target segments	50.5 (24.3–96.6)	52.5 (20.5–94.3)	50.5 (24.8–96.4)	0.919
Time (seconds) reading non-target segments during initial reading	0.0 (0.0–0.0)	0.0 (0.0–0.0)	0.0 (0.0–0.0)	0.795
Time (seconds) spent on non-target segments during answering the quiz questions	49.7 (22.0–90.8)	51.1 (17.5–94.3)	48.3 (23.6–85.2)	0.957

^a^The results for questions that had target segments (SoF table cells) relevant for quiz questions (Q2 and Q4 to Q7). Number of predetermined target segments for each question: Q1–none (cells with outcomes in the first table column of the SoF table were visible at all times); Q2–all cells under table columns “Placebo or standard care alone” or “Risk difference with remdesivir”; Q3–none (segment is visible at all times); Q4–all cells except for those in the table column “No of participants (studies)”; Q5–the cell in the table column “No of participants (studies)” and the row “All-cause mortality up to day 28”; the cell in the table column “Certainty of the evidence (GRADE)” and the row “Adverse events (any grade) at up to day 28”; Q7–all of the cells in the table column “Comments”.

^b^For 6 out of 7 questions (Q7 was excluded as it was not rated as true or false).

^c^Initial reading is the time spent from the opening the quiz to the opening the first question.

**Table 2:** Interactions per question (median, interquartile range) and percentage of correct answers about Summary of Findings (SoF) Table.

Question^a^	Number of times navigating between table and question	Number of table segments visited^b^			Percentage of correct answers (N = 121)
		Total	Target	Non-target	
Q1: Which outcome do you think is the most important one?	4 (2–6)	7 (0–21)	0 (0–0)	7 (0–24)	57
Q2: Which outcome has the biggest improvement with the intervention?	5 (3–7)	25 (8–39)	15 (5–22)	7 (1–18)	36
Q3: What is the setting for patients included in this evidence assessment?	1 (1–3)	0 (0–0)	0 (0–0)	0 (0–1)	93
Q4: Based on the information from this Summary of Findings Table, how would you formulate a recommendation for clinical practice?	4 (2–6)	16 (5–28)	9 (0–19)	6 (0–13)	38
Q5: How many participants were there in trials that assessed death as an outcome?	2 (1–3)	5 (2–12)	2 (1–2)	4 (1–10)	83
Q6: Why was the quality of evidence for the outcome of adverse events graded as very low?	3 (1–5)	7 (2–12)	2 (0–4)	5 (1–9)	60
Q7: Now look at the “Comments” column. Are the comments different from your recommendation? Please explain how	1 (1–2)	9 (2–14)	7 (0–12)	0 (0–3)	N/A^c^
Total	23 (15–30)	87 (54–129)	39 (22–54)	49 (27–77)	61

^a^Target SoF table segments: Q1–none (cells with outcomes in the first table column of the SoF table were visible at all times); Q2–all cells under table columns “Placebo or standard care alone” or “Risk difference with remdesivir”; Q3–none (segment is visible at all times); Q4–all cells except for those in the table column “No of participants (studies)”; Q5–the cell in the table column “No of participants (studies)” and the row “All-cause mortality up to day 28”; the cell in the table column “Certainty of the evidence (GRADE)” and the row “Adverse events (any grade) at up to day 28”; Q7–all of the cells in the table column “Comments”.

^b^The number of segments visited could be greater than the number of table segments, as students could click multiple times on the same table cell.

^c^Q7 had no specific knowledge question and was not scored.

Table 2 shows the number of times the students navigated between the table and the question, the number of segments visited for a specific question, and the percentage of correct answers. Overall, they often navigated between the table and the quiz question field and had many interactions with SoF table segments, with a similar number of visits to target and non-target segments. They correctly answered 61% of the questions.

Question Q2, which concerned the outcome with the largest improvement after the health intervention, had the lowest number of correct answers. That was also the question for which students visited the highest number of SoF table segments. For other questions, the number of visits to the target table segments was lower, although the students still visited several non-target table segments while answering the questions. Only the last question (Q7), which instructed them to look at a specific table segment (Comments) did not have visits to non-target segments.

The question about the study setting for studies (Q3) had most correct answers and almost no interaction with the SoF table, as this information was visible at all times in the SoF table.

Question Q4, which asked whether they would recommend the use of the intervention, had the second lowest test score, with only 38% (n = 46) correct answers. 23% of the students (n = 52) provided answers that that did not contain any recommendation. There was no difference in the answers the students from the study programmes in Croatian and English (χ^2^ = 0.011, P = 0.917).

For Q7, the students had to comment on the recommendation they wrote in Q4, after the Comment column of the SoF table became accessible for reading (i.e. it opened after a click on the segment). Many students (56%, n = 68) considered that there was no difference between their recommendation and the comment in the SoF table. About a third (30%, n = 36) noticed and correctly identified that their recommendation did not match the comment in the SoF table. There was no difference in the answers between the students from the study programmes in Croatian and English (χ^2^ = 5.797, *P* = 0.055).

Table 3 shows the times spent on individual tasks for each quiz question. The students spent more time on questions that required assessing the information in the SoF table before providing an answer. The examples include Q2 (the outcome with the largest improvement; median 231.7 s, IQR182.0–295.9 s), Q4 (providing a recommendation; median 277.3, IQR 159.6–391.4 s), Q5 (question about a specific outcome; median 49.6 s, IQR 31.3–87.7 s), Q6 (opinion about grading the evidence; median 108.5 s, IQR 69.68–144.6 s) and Q7 (comparison of the Comment table cell and own recommendation; median 144.1, IQR 69.1–222.5 s), compared to questions that had answers readily available, such as Q3 (setting for the patients; median 49.6 s, IQR 31.3–87.7 s). The students spent a lot of time answering Q1 (most important outcome in the SoF table; median 278.8 s, IQR 165.6–369.1 s), for which the answer in the table segments was visible at all times. For this question, the students spent most time reading non-target cells (median 96.1 s, IQR 0.0–159.1 s). For all other questions, the time spent reading non-target cells was more than three times shorter. In contrast, all measured times for Q3 (setting for the patients), which also had the information presented in non-blurred table cells, were much smaller.

**Table 3:** Time (seconds; median and interquartile range) spent on individual quiz actions for each question (n = 121).

Question^a^	Total time spent on a question, between the first opening and quiz submission	Time spent performing tasks^a^	Time reading all table segments during answering a question	Time reading target table segments during answering a question^b^	Time reading non-target table segments during answering a question
Q1: Which outcome do you think is the most important one?^c^	278.8 (165.6–369.1)	29.56 (19.0–51.7)	96.1 (0.0–159.1)	–	96.1 (1.7–175.4)
Q2: Which outcome has the biggest improvement with the intervention?	231.7 (182.0–295.9)	43.6 (24.0–65.4)	114.2 (73.4–172.4)	82.7 (38.8–132.7)	22.9 (2.8–49.7)
Q3: What is the setting for patients included in this evidence assessment?^c^	49.6 (31.3–87.7)	9.1 (6.6–21.9)	0.0 (0.0–0.0)	–	0.0 (0.0–8.1)
Q4: Based on the information from this Summary of Findings Table, how would you formulate a recommendation for clinical practice?	277.3 (159.6–391.4)	105.8 (64.4–172.1)	72.8 (30.3–144.9)	35.0 (0.0–77.6)	29.6 (0.0–59.4)
Q5: How many participants were there in trials that assessed death as an outcome?	94.2 (49.8–139.4)	12.3 (6.5–27.7)	34.6 (10.0–72.1)	7.3 (1.2–20.6)	18.2 (3.0–51.3)
Q6: Why was the quality of evidence for the outcome of adverse events graded as very low?	108.5 (69.6–144.6)	40.7 (29.4–55.1)	29.0 (14.7–51.0)	12.5 (0.0–20.2)	14.5 (5.4–35.0)
Q7: Now look at the “Comments” column. Are the comments different from your recommendation? Please explain how	144.1 (69.1–222.5)	64.7 (31.6–105.0)	36.7 (8.7–73.7)	29.3 (0.0–61.7)	0.0 (0.0–8.1)

^a^Target SoF table segments: Q1–n one (cells with outcomes in the first table column of the SoF table were visible at all times); Q2–all cells under table columns “Placebo or standard care alone” or “Risk difference with remdesivir”; Q3–none (segment is visible at all times); Q4–all cells except for those in the table column “No of participants (studies)”; Q5–the cell in the table column “No of participants (studies)” and the row “All-cause mortality up to day 28”; the cell in the table column “Certainty of the evidence (GRADE)” and the row “Adverse events (any grade) at up to day 28”; Q7–all of the cells in the table column “Comments”.

^b^Total time for actions of clicking on the table cells or changing between the table and question screen.

^c^For these questions there were no target table cells as the answers were in the unblurred parts of the SoF table.

**Figure 1:**
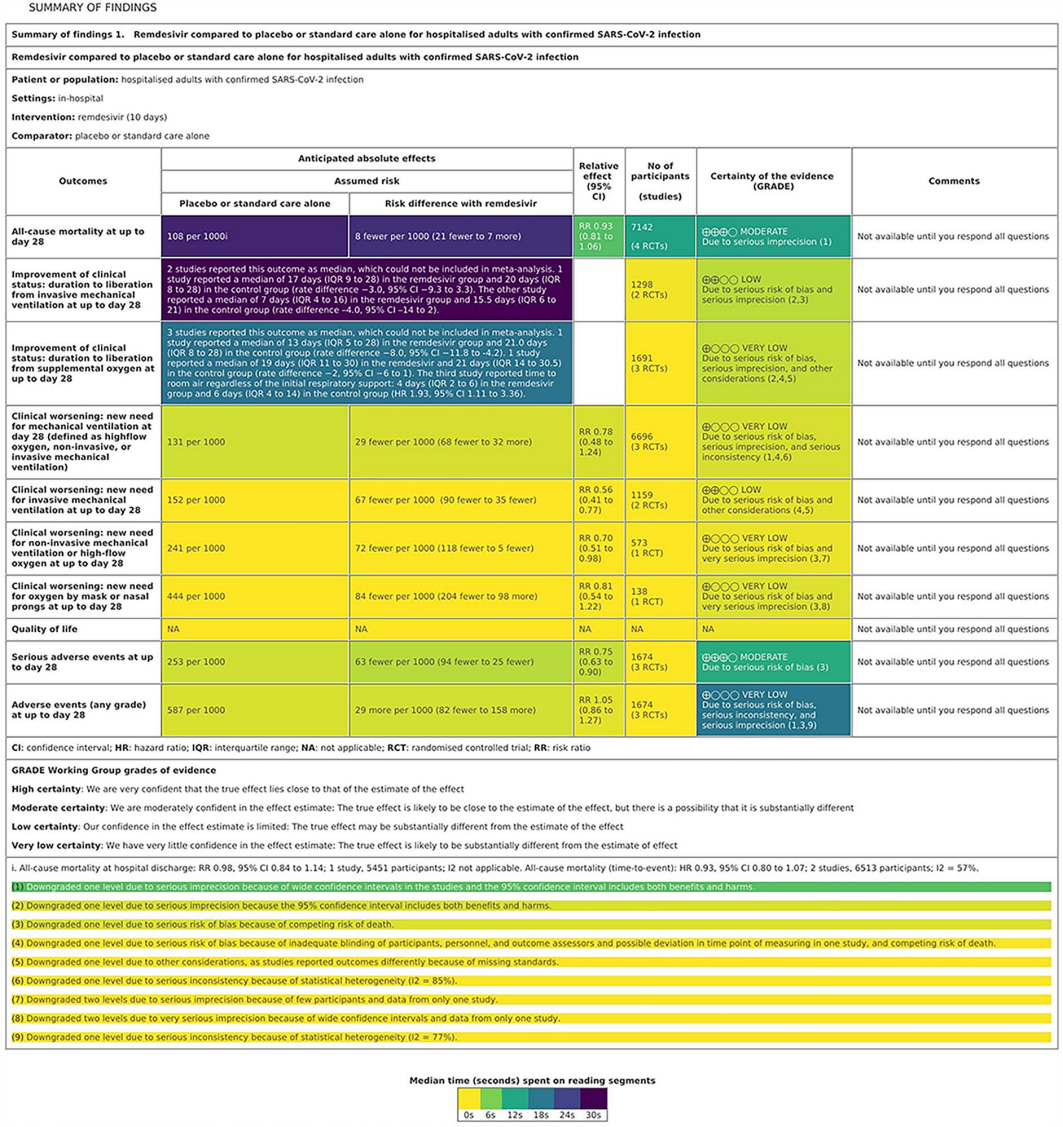
Heat map of reading times per Summary of Findings (SoF) Table elements while answering the first six quiz questions. The colours correspond to the median time spend on the individual table cell, from 0 (yellow) to 30 s (purple).

The heatmap of the SoF table for the first six questions (Fig. 1) showed that the students spent most time on table cells with the information on the observed results for the outcomes related to the mortality and to the improvement of clinical status related to discontinuation of mechanical ventilation (median 25.4 s, IQR = 16.8–34.3 s), followed by the table cells with the information on the improvement of clinical status related to supplemental oxygen (median 15.7 s, IQR = 9.2–26.5 s). These cells were relevant for answering Q3 (setting for patients), which concerned the largest change in relative effect. Although other cells in the table columns related to anticipated absolute effects were also target cells for that question, there was less interaction with them, up to a median of 2.7 s, IQR = 1.3–4.0 s. For Q5, about the number of patients in trials that assessed death as the outcome, the relevant cell was visited the most often (median 10.8 s, IQR = 8.3–13.9 s), but 4 other cells in that row were also visited for a median of 0.5 (IQR = 0.0–1.6) s. For Q6, about very low quality of evidence for adverse events, the students spent most time on the appropriate table cell (median 15.4, IQR = 11.1–19.4 s). Other cells in that table column, on the certainty of evidence, were also opened, although not relevant for Q6 or other questions. The students also opened the SoF table footnotes (Fig. 1), mostly about the certainty of evidence for all-cause mortality up to day 28.

**Figure 2:**
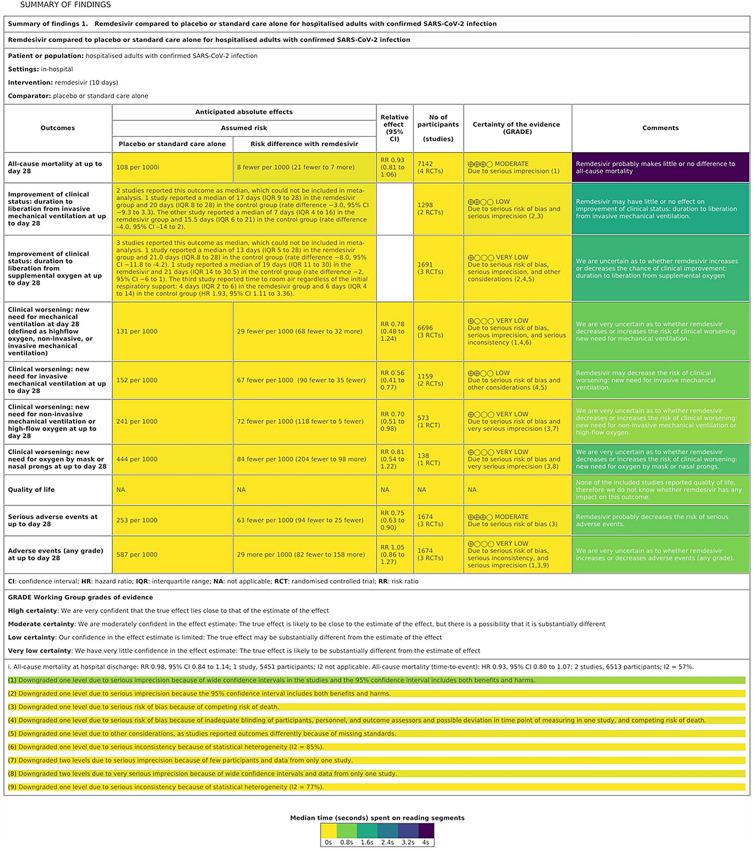
Heat map of reading times per Summary of Findings (SoF) Table elements while answering quiz question 7. The colours correspond to the median time spend on the individual table cell, from 0 (yellow) to 4 s (purple).

For Question 7, which required the students to compare the comments in the SoF table and their own recommendation, the heatmap of the table showed that students interacted only with the comments column, and did not open other cells in the table (Fig. 2). They also spent less time on individual table cells than for other questions. All comment cells were opened, and students spent a median of 0 to 4 s on them. The most visited cell in that table column was the one related all-cause mortality at up to day 28, on which students spent a median of 3.8 s, IQR = 3.1–5.0 s reading it. They also spent similar time interacting with the table footnote related to this comment cell.

## Discussion

To the best of our knowledge, this is the first study to explore how informed readers navigate through the SoF table to get answers to questions regarding an aspect of the table. As expected, the easiest question to answer in our study, as judged by the highest percentage of correct answers, total time expended in question and the number of segments visited during search for the given question, was the setting for patients in the study. It was a simple question, with the answer visible in the PICO table segment, without a need for clicking on blurred table cells. In contrast, the question asking for an evaluation of outcomes had the fewest correct answers, with the largest number of segments visited while searching for the answer. Long total times and especially times spent on non-target table cells for Q1 (most important outcome), which had the information in non-blurred table cells, visible at all times, also indicate that the students needed some time to explore the experimental environment and get used to a new quiz type. Judging from the time spent on certain table cells, students were mostly answer-oriented, but also took their time to get familiar with the other aspects of table, irrelevant of their quiz task. The heatmap of the SoF table confirmed students’ focus on answering a specific quiz question. These results also support the idea that SoF tables may be well constructed for finding answers to clinical questions. Based on the number of cells visited, the students used the uppermost table cells for orientation: the cells for the first three outcomes were most visited cells, whereas the cells for the quality of life outcomes were either not visited or rarely visited.

The strength of our study is the use of the Read&Learn tool as an objective follow-up of the critical thinking process during the interpretation of an SoF table. We also used open-ended answers which have been shown to better reflect students’ knowledge than multiple-choice questions with a single correct answer^12^. In order to ensure independent assessment of the question responses for all quiz questions, open-ended answers were separately assessed by two assessors. Compared to the eye-tracking approach^13^, the strength of our methodological approach is that it clearly indicated with which parts of the SoF table the students interacted with, as just having eye set on the table element does not necessarily mean that the person is reading that element.

The study limitations include the study sample, which was geographically restricted to a single medical school in a single country. This does not allow generalizations to other geographical and professional settings.

The students performed worst on the questions that required critical thinking and assessment of the information in SoF table cells, which is expected from third-year medical students. This finding is not surprising, as current medical curricula have been shown to often lack effective promotion of critical thinking^14,15^. They may not have had sufficient time and clinical experience to successfully master the SoF table, although the topic of COVID-19 vaccine trial was not complicated and was familiar and engaging for medical students. The importance of clinical experience is at least partially supported by the finding that students from the medical program in Croatian had better overall quiz scores that the students from the program in English, because the former group had the course later than the latter, having more time to acquire relevant knowledge from clinical courses in the third year of medical studies. Learning to memorize facts, understand evidence-based concepts and apply the knowledge in familiar situations, are considered lower-level educational objectives^16^. The students may not have sufficient expertise and experience for higher cognitive educational outcomes required for understanding and applying SoF tables in practice, such as in Q4 (students’ own recommendations for practice based on the information from the SoF table). These higher cognitive outcome levels include *evaluating* (ability to make judgments about the value of ideas or materials, and/or compare different ideas); and *creating* (ability to build a new structure or pattern from diverse elements)^17,18^.

It is difficult to provide a full theoretical or practical interpretation of our findings, as there are no similar studies using the Read&Learn tool in evidence-based medicine. In psychology, the tool was used to observe how thinking out loud affects searching for task-relevant information in a presented text^19^, and to prove that adjunct questions influence student text processing^20^. In mathematics, the reading time of students aged 15–16 years for arithmetic problems was indicative of a problem’s complexity^9^. This was confirmed in our study, where the quiz questions that required processing and assessing the information in the SoF table rather than just finding the correct number or a statement, took longer time and more interactions with the table cells. The results from our study cannot be generalized to all medical students. As there was not difference in the interaction with the SoF table segments for the students attending the Croatian and the English study programmes, it seems that the knowledge of English was not a barrier to the understanding of an SoF table and answering knowledge questions about the information presented in the table.

Our study opens a new line of research that may help stakeholders in health evidence synthesis with producing information in formats optimal for quick and effective use of health evidence in practice. In order to make outcomes important for clinical decision more prominent, the organization of evidence synthesis in an SoF table seems to be important. For example, the quality of life outcome in the tested SoF was placed at the bottom of the table, together with adverse events data and was rather ignored by the participants. The current SoF table format may be suitable even for beginners for orientation and finding of specific information, but the format and distribution of the table cells may be important for more mindful processing of SoF table information in translating the evidence to practice. Further developments of the tool and metrics for understanding the complexity of health information in evidence-based medicine and cognitive processes during the assessment of this information may help develop teaching and learning paths for health care professionals.

## Methods

The study was conducted at the University of Split School of Medicine (USSM) during the mandatory, vertically integrated course Research in Biomedicine and Health (RBH), in which students learn the basics of research methodology and biostatistics^10^. In the third year of their medical studies, RBH III course introduces students to the principles of evidence-based medicine, where they learn how to navigate through research sources and how to read and critically asses health evidence at the start of their clinical curriculum.

### Participants and procedure

The study included students attending the last day of the RBH III course at the USSM. The students of the English study programme attended the course in October and November 2021 (start of 2021/2022 academic year), and the students from the Croatian study programme attended the course in June 2022 (end of 2021/2022 academic year). Aside from the teaching language, the two courses are identical, as per national programme accreditation, including the same teachers.

The last day of a five-day course included a seminar on clinical practice guidelines, which introduced the concept and structure of SoF tables as a method for clear and systematic presentation of study findings in a systematic review. The seminar was followed by a practical exercise, where the students had to answer questions about the evidence presented in a SoF table and make practical recommendation based on the evidence. The SoF table used in the study was on remdesivir compared to placebo or standard care alone for hospitalized adults with confirmed COVID-19 infection^11^.

We used the Read&Learn tool^9^ to assess how students navigated through the SoF table to answer questions about the evidence presented in the table. The students received a detailed oral explanation about the procedure before the quiz. When they opened the quiz on the screen, they were presented with two screen sections – one containing a question and a box for the answer, and the other one the SoF table with blurred table cells with data. Supplementary Fig. 1 presents the initial view of the SoF table with blurred cells and Supplementary Fig. 2 presents the question screen, which is opened by clicking on the questions button on the table screen. Students could alternate between the SoF table screen and the question until they wrote the answer for the question. The system then presents the second question and the same SoF table. The first table column with the outcomes and the headings of other table columns were visible at all times. Students were instructed to navigate and read relevant table cells before answering a specific question. The table cell content could be made visible by clicking on it. When a student clicked on another table cell to see its content, the previous cell got blurred. After answering a question, the screen presented the next question and the SoF table with all table cells blurred again. The table’s comment section and relevant footnotes were blurred at all times until the last question (Q7).

The students had to answer seven quiz questions with open-ended answers. All data needed for answering Questions 1 to 6 could be found in the target cells in the SoF table, and no additional calculations or information were required from students. For each question, we determined which table segments were target segments (see legend to Table 1); the remainder were considered to be non-target segments. Two investigators (JM and RT) independently scored the questions to assess whether they matched the information in the SoF table. There were no disagreements in their scores. Question 4 was scored separately from other questions as it addressed students’ opinions and not knowledge. We considered an answer to this question to be correct if a student’s recommendation matched the comment cell in the SoF table. Two assessors (JM and LU) independently scored the answers; no disagreements occurred in their scores.

After answering quiz Question 6 (quality of evidence for adverse events), the students had to click the “Finish this unit” button, which made the Comments column of the SoF table visible, so that they could compare their recommendations from Question 4 to those in the SoF table. Question 7 required the students to explain if and why their recommendation differed from the comment in the SoF table. We did not rate it, but we reported the number of students who stated that their recommendation corresponded to the Comments column in the SoF table.

### Ethical considerations

The study was performed in accordance with relevant regulations and guidance on social science research involving human participants. The Ethics Committee of the University of Split School of Medicine approved the study (document Class 003-08/19-03/0003, Reg. No. 2181-198-03-04-19-0044) as a part of the research grant funded by the Croatian Science Foundation (grant No. IP-2019-04-4882). Informed consent was obtained from the participants. The analysis used the anonymized data from the quiz.

### Measurements of the interaction with SoF table cells

The Read&Learn tool^9^ records all user interactions with the statements, questions and response options, as well as timestamps for the interactions. The SoF table used in the study had a total of 66 segments (table cells).

Question-level measurements: (1) Number of times navigating between text and question; (2) Number of segments (SoF table cells) visited during search for the given question; (3) Percentage of correct answers; (4) Total time (s) spent in the question, between first opening and validation; (5) Time (s) spent on performing actions (e.g. clicking on a table cell) for a question; (6) Time (s) reading segments during search for the given question, divided into the time reading target segments, and the time reading non-target segments (s).

Text level measurements: (1) Total time (s) in text, including reading and writing time; (2) Number of segments visited during the initial reading (e.g. the time before opening the quiz and opening the first question); (3) Time (s) reading target segments during the initial reading; (4) Time (s) reading target segments; (5) Time (s) reading non-target segments during the initial reading; (6) Time (s) reading non-target segments.

For questions which had target segments (Q2, Q4, Q5, Q6 and Q7), we calculated the median total times for these segments for each student. Median times (in seconds) spent of reading SoF table segments are also presented as heat maps (Figs. 1 and 2).

### Statistical analysis

We presented data as median and interquartile range (IQR). We analyzed the differences between the students from two study programs using the Mann–Whitney test, with Bonferroni correction for 13 table elements, which gave the *P*-value limit of 0.004. We performed the statistical analyses using the MedCalc Statistical Software, version 20.0.13.0. (Medcalc Software, Oostende, Belgium), and JASP software 0.16.3. (JASP team, Amsterdam, Netherlands, 2022).

### Supplementary Information

Supplementary Figures.

## Data Availability

The datasets generated during and/or analysed during the current study are available from the corresponding author on reasonable request.

## References

[1] Higgins, J. P. T. et al. Cochrane Handbook for systematic reviews of interventions version 6.3. Cochrane. www.training.cochrane.org/handbook (Accessed February 2022) (2022).

[2] Rosenbaum SE, Glenton C, Nylund HK, Oxman AD (2010). User testing and stakeholder feedback contributed to the development of understandable and useful summary of findings tables for cochrane reviews. J. Clin. Epidemiol..

[3] Rosenbaum SE, Glenton C, Oxman AD (2010). Summary-of-findings tables in cochrane reviews improved understanding and rapid retrieval of key information. J. Clin. Epidemiol..

[4] Yepes-Nuñez JJ (2018). Two alternatives versus the standard grading of recommendations assessment, development and evaluation (GRADE) summary of findings (SoF) tables to improve understanding in the presentation of systematic review results: A three-arm, randomised, controlled, non-inferiority trial. BMJ Open.

[5] Carrasco-Labra A (2015). Comparison between the standard and a new alternative format of the summary-of-findings tables in cochrane review users: Study protocol for a randomized controlled trial. Trials.

[6] Wolfe JM, Horowitz TS (2017). Five factors that guide attention in visual search. Nat. Hum. Behav..

[7] Yang Y, Mo L, Lio G, Huang Y, Perret T, Sirigu A, Duhamel JR (2023). Assessing the allocation of attention during visual search using digit-tracking, a calibration-free alternative to eye tracking. Sci. Rep..

[8] Spinner P, Gass S, Behney J (2013). Ecological validity in eye-tracking: An Empirical Study. Stud. Second Lang. Acquis..

[9] Sanz MT, López-Iñesta E, Garcia-Costa D, Grimaldo F (2020). Measuring arithmetic word problem complexity through reading comprehension and learning analytics. Mathematics.

[10] Buljan I, Tokalić R, Marušić M, Marušić A (2019). Health numeracy skills of medical students: Cross-sectional and controlled before-and-after study. BMC Med. Educ..

[11] Spinner CD (2020). Effect of remdesivir vs standard care on clinical status at 11 days in patients with moderate COVID-19: A randomized clinical trial. JAMA.

[12] Sam AH, Westacott R, Gurnell M, Wilson R, Meeran K, Brown C (2019). Comparing single-best-answer and very-short-answer questions for the assessment of applied medical knowledge in 20 UK medical schools: Cross-sectional study. BMJ Open.

[13] Mondal S, Pratim Das P, Bhattacharjee Rudra T (2022). Measuring code comprehension effort using code reading pattern. Sadhana.

[14] Huang GC, Newman LR, Schwartzstein RM (2014). Critical thinking in health professions education: Summary and consensus statements of the millennium conference 2011. Teach. Learn. Med..

[15] Richards JB, Hayes MM, Schwartzstein RM (2020). Teaching clinical reasoning and critical thinking: From cognitive theory to practical application. Chest.

[16] Buljan I (2021). Cognitive levels in testing knowledge in evidence-based medicine: A cross sectional study. BMC Med. Educ..

[17] Bloom BS (1956). Taxonomy of Educational Objectives: The Classification of Educational Goals.

[18] Anderson LW, Krathwohl DR (2001). A Taxonomy for Learning, Teaching, and Assessing: A Revision of Bloom’s Taxonomy of Educational Objectives.

[19] Manez I, Vidal-Abarca E, Magliano J (2022). Comprehension processes on question-answering activities: A think-aloud study. Electron J. Res. Educ. Psychol..

[20] Rubio A, Vidal-Abarca E, Serrano-Mendizábal M (2022). How to assist the students while learning from text? Effects of inserting adjunct questions on text processing. Instr. Sci..

