# Supplementary material for: Tool to assess recognition and understanding of elements in Summary of Findings Table for health evidence synthesis: a cross-sectional study
Source: Sci Rep. 2023 Oct 23;13:18044. doi: 10.1038/s41598-023-45359-x (PMC10593927; doi:10.1038/s41598-023-45359-x)

Matas et al. Tool to assess recognition and understanding of elements in Summary of Findings Table for health evidence synthesis: A cross-sectional study

Legend to the Supplementary Figures

Supplementary Figure 1. Computer screen presenting the initial view of the Summary of Findings table.

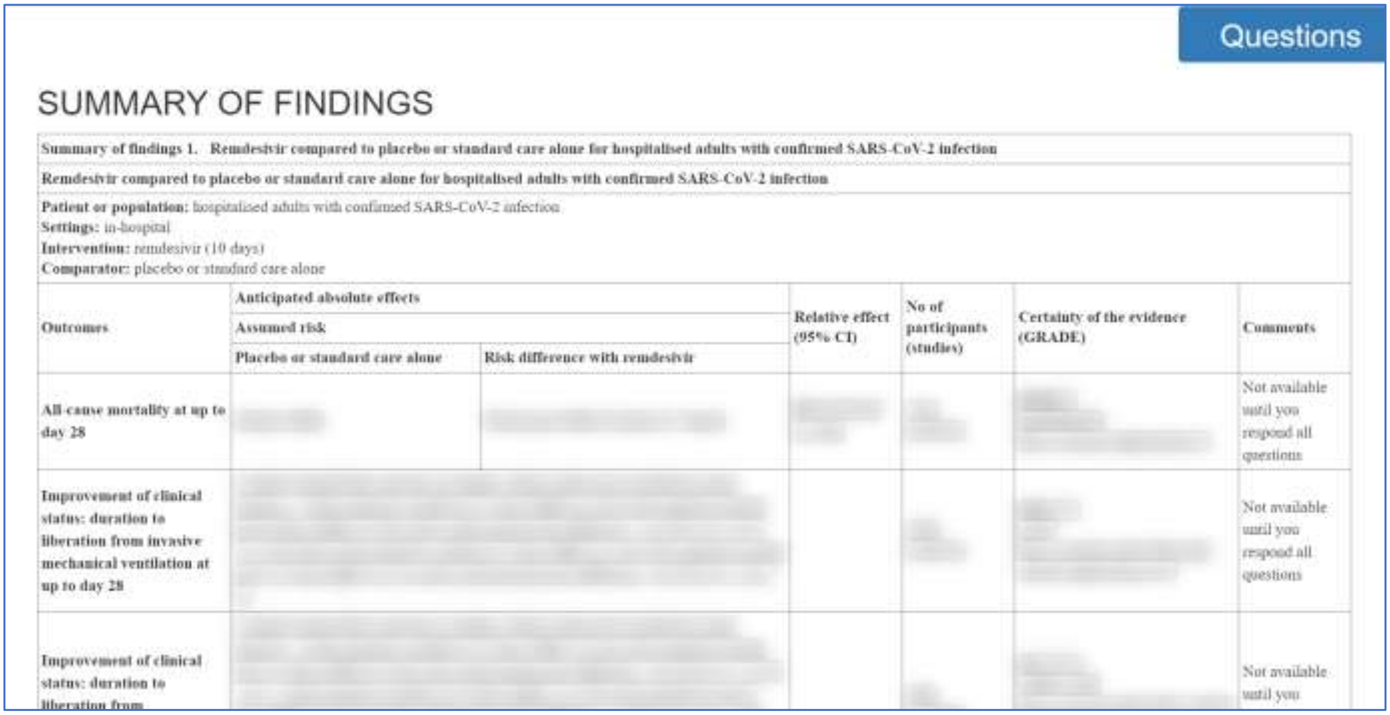

Supplementary Figure 2. Questions screen (Question 1 visible on screen).

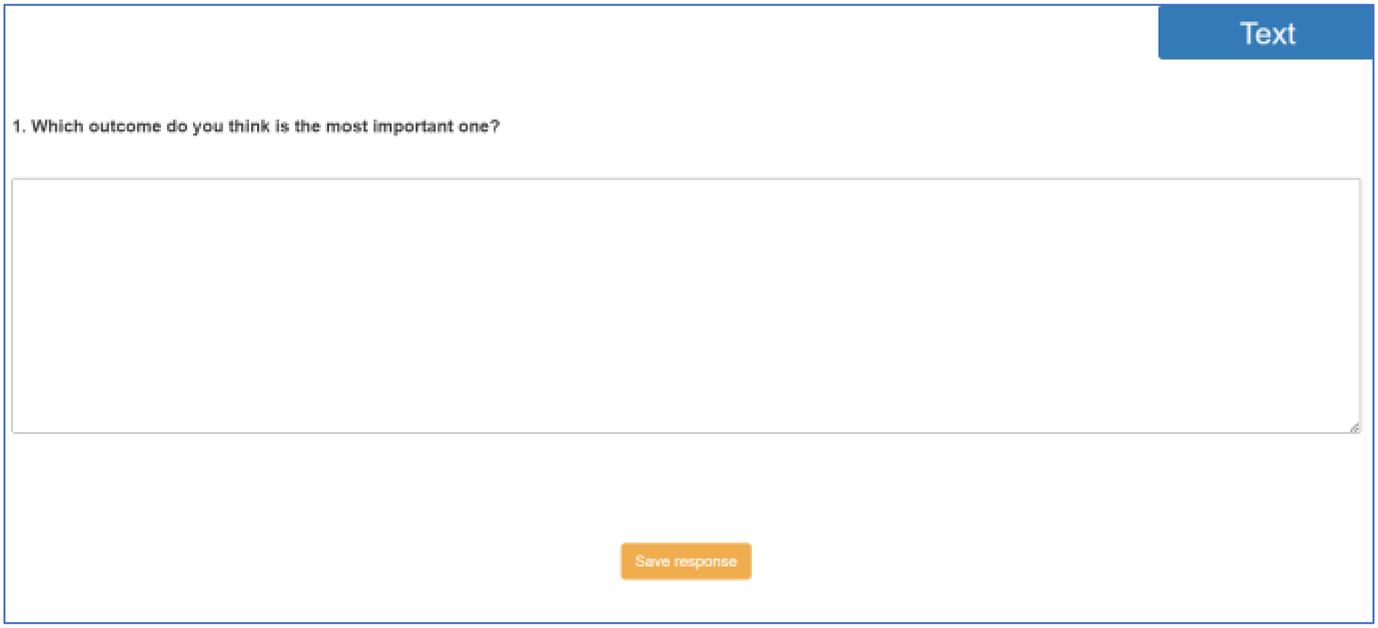

Supplement: Supplementary file 1 — Supplementary Figures. [file 41598_2023_45359_MOESM1_ESM.pdf]
